# RNA-Based Therapies for Lipoprotein(a) Reduction: Antisense Oligonucleotides, Small Interfering RNAs, and Emerging Platforms

**DOI:** 10.1007/s11883-026-01474-4

**Published:** 2026-10-14

**Authors:** Vibhu Parcha, Vera A. Bittner

**Affiliations:** Division of Cardiovascular Disease, Department of Medicine, University of Alabama at Birmingham, GSB 444, 521 19th Street South, Birmingham, AL 35233 United States

**Keywords:** Lipoprotein(a), Antisense oligonucleotide, Small interfering RNA, Gene editing, RNAi therapeutics, Cardiovascular disease

## Abstract

**Purpose of Review:**

Lipoprotein(a) [Lp(a)] is an independent, genetically determined, and causal risk factor for atherosclerotic cardiovascular disease (ASCVD) and calcific aortic valve stenosis. Elevated Lp(a) affects approximately 20% of the global population and is not meaningfully modified by lifestyle intervention or existing lipid-lowering pharmacotherapy. This review summarizes the biology of Lp(a) as a therapeutic target, evaluates the efficacy of currently available lipid-lowering agents against this target, and describes the mechanistic principles, clinical evidence, and unresolved questions associated with RNA-based and emerging small-molecule and gene-editing approaches to Lp(a) reduction.

**Recent Findings:**

Four RNA-directed agents targeting hepatic apolipoprotein(a) [apo(a)] synthesis have entered phase 2/3 development. This includes pelacarsen, an antisense oligonucleotide (ASO), and olpasiran, zerlasiran, and lepodisiran, which are N-acetylgalactosamine (GalNAc)-conjugated small interfering RNAs (siRNAs). Phase 2 data demonstrate Lp(a) reductions ranging from 79% to approximately 97% on a placebo-adjusted basis, approaching the practical limit of apo(a) suppression, with favorable short-term safety profiles. A fifth, structurally distinct siRNA conjugated with GalNAc at both strand termini has recently completed first-in-human testing, sustaining reductions up to 97% through 48 weeks after a single dose without injection-site reactions. Recently, the topline results of phase 3 Lp(a) HORIZON (pelacarsen) were released and demonstrate a substantial Lp(a) reduction without meeting the trial’s primary composite cardiovascular endpoint. Two further outcomes trials, OCEAN(a)-Outcomes (olpasiran) and ACCLAIM-Lp(a) (lepodisiran), remain ongoing. An oral small molecule, muvalaplin, achieved comparable reductions in phase 2 testing. Lastly, early non-human primate data, together with a completed phase 1 trial of an analogous CRISPR-Cas9 construct targeting a related hepatic lipid gene, support the feasibility of a CRISPR-Cas9-based gene-editing approach capable of durable apo(a) suppression from a single administration.

**Summary:**

The pharmacology of Lp(a) suppression is now well characterized across three distinct therapeutic modalities. Whether the magnitude of biochemical reduction observed translates into a meaningful and safe reduction in cardiovascular events across different populations remains undetermined and will be addressed by the ongoing outcomes trials.

## Introduction

Lipoprotein(a) [Lp(a)], first described by Berg (1963), is a structurally distinct lipoprotein particle composed of a low-density lipoprotein (LDL)-like core whose apolipoprotein B-100 (apoB) moiety is covalently linked to apo(a), a highly glycosylated, plasminogen-like protein. For several decades, the clinical significance of Lp(a) was obscured by assay variability and the absence of any modifying therapy. Mendelian randomization (MR) and genome-wide association studies have since established Lp(a) as a causal, genetically determined, and independently acting risk factor for ASCVD (atherosclerotic cardiovascular diseases) and calcific aortic valve stenosis [1, 2], prompting the development of a new class of RNA (ribonucleic acid)-directed therapeutics.

Elevated Lp(a) affects an estimated 20% of the global population [3]. Lp(a) concentration is determined by more than 90% of the variance at the *LPA* gene locus and is not appreciably modified by diet, exercise, or conventional lipid-lowering therapy. Among existing agents, statins do not meaningfully or consistently lower Lp(a), whereas PCSK9 inhibitors achieve only a 20–30% reduction, an effect generally considered insufficient to confer independent clinical benefit in patients with very high baseline concentrations [3–9]. The therapeutic need for a more potent Lp(a)-lowering strategy has therefore been long established. The *LPA* transcript is expressed almost exclusively in hepatocytes, a tissue readily targetable by GalNAc-conjugated oligonucleotides. By silencing *LPA* mRNA through RNase H-mediated degradation (the ASO mechanism) or RNA-induced silencing complex [RISC]-mediated cleavage (the siRNA mechanism), these agents achieve reductions in circulating Lp(a) substantially exceeding those of any previously available therapy and with effect sustained for weeks to months after a single subcutaneous injection.

This review summarizes the biology of Lp(a) as a drug target, evaluates the limited efficacy of currently available lipid-lowering therapies, describes the pharmacological principles of hepatocyte-directed RNA silencing, the clinical evidence for RNA-based, small-molecule, and gene-editing agents, and safety considerations. In light of the newly reported Lp(a) HORIZON topline results and the two ongoing outcomes trials, we examine which questions remain unresolved.

## Lipoprotein(a): Structure, Genetics, and Pathophysiology

### Molecular Structure and Assembly

The Lp(a) particle consists of a single apoB-100 molecule covalently bound via a disulfide bridge to apo(a). The apo(a) component is encoded by the *LPA* gene on chromosome 6q26-6q27 and shares approximately 70–80% amino acid sequence homology with plasminogen and forms the molecular basis of the prothrombotic properties that distinguish Lp(a) particles from LDL particles [10].

The plasma concentration of Lp(a) is principally determined by a copy-number variant within the *LPA* gene, the kringle IV type 2 (KIV-2) repeat, which spans up to 70% of the coding sequence and generates on the order of 40 distinct alleles and approximately 1,600 possible genotypes [11]. The low molecular weight isoforms (10–22 KIV-2 repeats) produce concentrations four to five times higher than high molecular weight isoforms, reflecting more efficient secretion of the smaller protein. Isoform size accounts for only 40–70% of Lp(a) variance, it is not fully predictive of Lp(a) concentration at the individual level. Alleles of identical size can differ in concentration by as much as 200-fold largely due to splice-site variants within the KIV-2 region. Two such variants, 4925G > A and 4733G > A, reduce isoform-adjusted Lp(a) by approximately 31 mg/dL and 13 mg/dL, respectively, and together constitute the largest genetic modifiers of Lp(a) concentration after isoform size [11]. This inverse relationship between isoform size and concentration also has implications for assay behavior. Molar-based assays (nmol/L), which report apo(a) particle number, are less susceptible to isoform-related inaccuracy than mass-based assays (mg/dL), a distinction with potential consequences for both epidemiological risk thresholds and therapeutic monitoring, although a post hoc analysis comparing mass-based, molar-based, and mass spectrometry assays in ODYSSEY OUTCOMES found similar percentage reductions and equal prognostic value across methods when Lp(a) was compared by percentile of the baseline distribution [8]. Beyond isoform size, specific single nucleotide polymorphisms at the *LPA* locus further modulate plasma Lp(a) concentration, with rs10455872 and rs3798220 associated with small apo(a) isoforms and elevated Lp(a) [11].

### Mechanisms of Cardiovascular Risk

The atherogenicity of Lp(a) is multifactorial. Its LDL-like core drives foam cell formation and plaque development in a manner analogous to but mechanistically distinct from LDL. On a per-particle basis, MR analyses indicate that Lp(a) is considerably more atherogenic than LDL-cholesterol. However, this comparison should be interpreted against the observation that Lp(a) particles constitute only approximately 3% of all apoB-containing lipoproteins in the general population and rises to at most 15% in the highest Lp(a) decile [12].

The apo(a) component confers additional risk through two principal pathways. First, Lp(a) is the dominant plasma carrier of oxidized phospholipids which amplify vascular inflammation through activation of innate immune pathways and promote osteogenic differentiation of valvular interstitial cells and serve as the likely mechanistic link to calcific aortic valve stenosis. Second, the structural homology between apo(a) and plasminogen allows Lp(a) to compete with plasminogen for binding sites on fibrin and endothelial cell surfaces, inhibiting fibrinolysis, and promoting a prothrombotic state that contributes to the excess risk of myocardial infarction (MI) and stroke associated with elevated Lp(a) independent of LDL-cholesterol.

### Epidemiology and Guideline Thresholds

Population studies have demonstrated a continuous, approximately log-linear relationship between Lp(a) concentration and ASCVD risk without an apparent threshold. Individuals with Lp(a) of 50, 100, and 150 mg/dL face approximately 1.40-, 1.95-, and 2.72-fold higher ASCVD risk, respectively, compared with those at the population median [13]. Among adults with established ASCVD, 13–20% have Lp(a) concentrations of 150 nmol/L or higher, defining a substantial secondary-prevention population plausibly eligible for future Lp(a)-directed therapy.

The 2026 AHA/ACC Guideline on the Management of Dyslipidemia [14], which retires and replaces the 2018 AHA/ACC Guideline on the Management of Blood Cholesterol [15], now gives measurement of Lp(a) a Class 1 recommendation. In all adults, Lp(a) concentration should be measured at least once for ASCVD risk assessment [14]. The guideline recommends use of Lp(a) as a “semi-quantitative” risk enhancer. The guideline notes that an Lp(a) of 430 nmol/L (180 mg/dL) confers roughly 4-fold higher risk, which is comparable to the risk associated with heterozygous familial hypercholesterolemia. The guideline further recommends cascade testing of first-degree relatives when an individual has familial hypercholesterolemia, premature ASCVD, or high Lp(a) (Class 1). The 2025 ESC/EAS focused update similarly recommends one-time Lp(a) measurement in adulthood [16], and the National Lipid Association has issued consistent guidance supporting opportunistic screening [17]. Neither the 2026 AHA/ACC guideline nor its international counterparts can recommend a specific Lp(a)-lowering pharmacotherapy, since none has regulatory approval, underscoring the gap that the RNA-based, small-molecule, and gene-editing agents discussed below are intended to close.

## Limitations of Existing Lipid-Lowering Therapies

The rationale for developing an entirely new therapeutic class for Lp(a) reduction rests on the observation that no currently available lipid-lowering agent produces a clinically meaningful and durable reduction in Lp(a) (Table 1). The effect of statins on Lp(a) is heterogeneous across trials: a patient-level meta-analysis of seven statin outcome trials found a pooled percentage change of -0.4% (95% CI, -7 to 7), with three trials showing a mean increase of 2% to 15% and four trials showing a mean decrease of 1% to 13% [9]. This finding has been corroborated by a subsequent systematic review and meta-analysis [7]. The 2026 AHA/ACC dyslipidemia guideline separately notes that statins may modestly increase Lp(a) in some individuals, with a mean absolute difference of 1.1 mg/dL compared with placebo, and states explicitly that statin therapy should not be withheld for fear of increasing Lp(a) given its well-established cardiovascular benefit [14]. This point carries direct clinical relevance, since patients occasionally discontinue statin therapy specifically out of concern that it will raise their Lp(a). Ezetimibe produces only a marginal reduction of uncertain clinical significance. Niacin lowers Lp(a) by approximately 23% in meta-analyses, but the AIM-HIGH trial demonstrated no cardiovascular benefit from niacin even among participants with Lp(a) above 125 nmol/L at baseline [18].

**Table 1:** Effect of currently available lipid-lowering therapies on lipoprotein(a)

Agent	Mechanism	Route/Frequency	Effect on Lp(a)
Statins	HMG-CoA reductase inhibition	Oral, daily	Heterogeneous effect across trials; pooled change − 0.4% (95% CI, -7 to 7) in a patient-level meta-analysis of 7 trials [7, 9]; guideline-cited mean absolute increase of 1.1 mg/dL vs. placebo[14]
Ezetimibe	Cholesterol absorption inhibition	Oral, daily	0–7% reduction of uncertain clinical significance
Niacin	Reduced apoB synthesis	Oral, 2–3 times daily	Approximately 23% reduction; no ASCVD benefit demonstrated even among participants with Lp(a) > 125 nmol/L[18]
PCSK9 monoclonal antibodies	Upregulation of hepatic LDL receptors	SC injection, 1–2 times monthly	20–30% reduction. Evolocumab: median 26.9%, attenuated to 16% in the highest Lp(a) quartile[5]. Alirocumab: median 23% [19]
Inclisiran	siRNA targeting hepatic PCSK9 mRNA	SC injection, biannual	17–29% reduction, an incidental consequence of LDL receptor upregulation rather than direct apo(a) synthesis inhibition[21]
Enlicitide	Oral macrocyclic peptide; inhibits PCSK9 binding to LDL receptors	Oral, daily	28.5% reduction[20]
CETP inhibitors	Reduced apo(a) production	Oral, daily (investigational)	Anacetrapib: 25% reduction, with cardiovascular benefit consistent with non-HDL-cholesterol lowering alone[23]. Obicetrapib: 33.5% reduction [24]
Lipoprotein apheresis	Mechanical particle removal	Every 1–3 weeks	70–80% reduction after a single session; the most effective current option but limited by cost, access, rebound between sessions and lack of randomized data showing benefit on ASCVD reduction [25]

The PCSK9 monoclonal antibodies are the most Lp(a)-active approved therapy. In the FOURIER trial, evolocumab produced a median 26.9% Lp(a) reduction, attenuated to only 16% among participants in the highest Lp(a) quartile [5]. In ODYSSEY OUTCOMES, alirocumab produced a median 23% reduction [19]. A subsequent analysis comparing mass-based, molar-based, and mass spectrometry Lp(a) assays in this trial found similar percentage reductions and equal prognostic value across assay methods [8]. Multivariable modeling suggested a 2.7% reduction in major adverse cardiovascular events per 5 mg/dL reduction in on-treatment Lp(a), independent of the accompanying change in non-HDL-cholesterol [19]. Neither trial could statistically confirm that the observed cardiovascular benefit was attributable specifically to Lp(a) lowering. However, both trials observed greater reduction in major adverse cardiovascular events among participants with higher, as opposed to lower, baseline Lp(a). The 2026 ACC/AHA guideline gives a Class 1 recommendation for use of PCSK9 monoclonal antibodies in individuals with clinical ASCVD and elevated Lp(a) who have not achieved LDL-C and non-HDL-C treatment goals on maximally tolerated statin therapy [14].

Enlicitide, an oral macrocyclic peptide that blocks PCSK9 from binding to LDL receptors, produced a 28.5% reduction in Lp(a) in its pivotal phase 3 trial, an incidental effect of its primary LDL-cholesterol-lowering mechanism analogous to that of the injectable PCSK9 inhibitors [20]. Since the trial enrolled an unselected hypercholesterolemia population rather than one selected for elevated Lp(a), and percentage Lp(a) reduction with PCSK9-pathway agents is generally larger at lower baseline concentrations, as observed with evolocumab in FOURIER [5], the modest absolute Lp(a) reduction likely achieved in most participants should temper enthusiasm for this percentage figure in isolation.

Inclisiran is an approved GalNAc-conjugated siRNA administered twice yearly that lowers Lp(a) by 17–29% [21] as an incidental consequence of PCSK9 silencing and consequent upregulation of hepatic LDL receptor activity rather than direct suppression of apo(a) synthesis with a pooled analysis of the ORION-10 and ORION-11 trials reporting a similar range [22]. Cholesteryl ester transfer protein (CETP) inhibitor Anacetrapib lowered Lp(a) by 25% in the REVEAL trial, though the accompanying cardiovascular benefit was of a magnitude consistent with non-HDL-cholesterol lowering alone [23]. In phase 3, CETP inhibitor obicetrapib produced a 33.5% reduction among participants with a median baseline Lp(a) of approximately 40 nmol/L [24]. Neither of these agents have regulatory approval. Lipoprotein apheresis remains the most effective current option. It achieves a 70–80% reduction after a single session [25], but its impracticality, requiring weekly to every-three-week sessions with venous access, and the lack of randomized ASCVD-outcome data underscore the need that RNA-based therapy with quarterly to semiannual dosing is intended to address.

## RNA-Based Therapeutic Platforms: Antisense Oligonucleotides and siRNAs

### The GalNAc Delivery Paradigm

The liver-restricted expression of *LPA* mRNA renders this target well suited to hepatocyte-directed RNA therapy which is delivered through N-acetylgalactosamine (GalNAc). GalNAc is a triantennary sugar ligand with high affinity for the asialoglycoprotein receptor, which is constitutively and abundantly expressed on the hepatocyte sinusoidal surface. Following subcutaneous injection, GalNAc-conjugated oligonucleotides undergo rapid, receptor-mediated endocytosis in the liver. After endosomal escape, the payload engages the cytoplasmic or nuclear silencing machinery. GalNAc conjugation has reduced required therapeutic doses by 10- to 30-fold relative to unconjugated oligonucleotides and improved the specificity and duration of hepatocyte gene silencing [26].

The empirical case for this modification is illustrated within the pelacarsen development program. The unconjugated predecessor molecule produced a 67–72% Lp(a) reduction after approximately three months but caused injection-site reactions in 10–19% of participants and influenza-like reactions in 10%. The GalNAc-conjugated successor, subsequently named pelacarsen, achieved greater potency without these adverse effects [27].

### Antisense Oligonucleotides

**Fig. 1:**
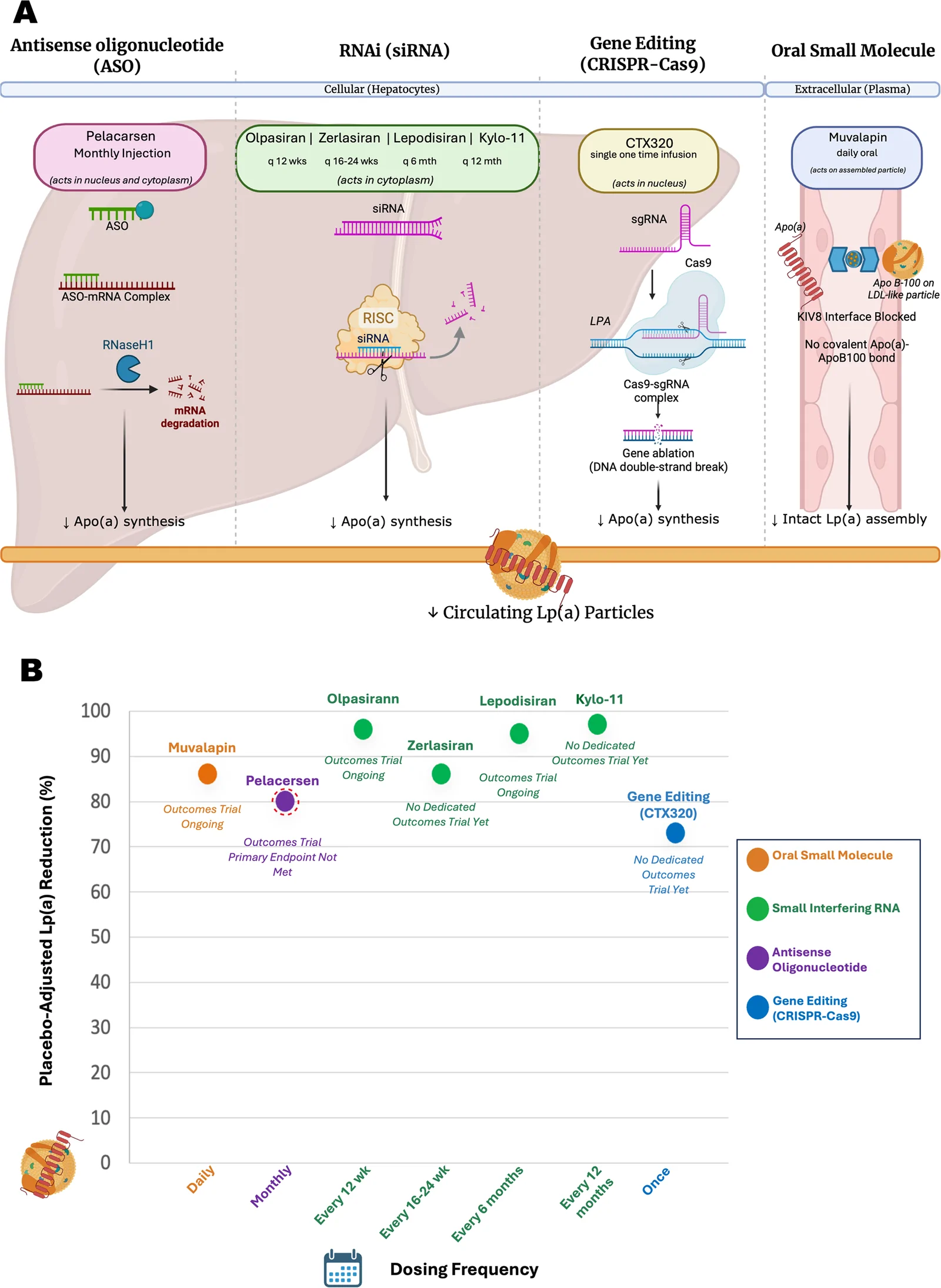
The Lipoprotein(a) Lowering Therapeutic Landscape Panel **A**: Demonstrates the mechanism of action of various intra-hepatic cellular and extra-hepatic non-cellular lipoprotein (a) [Lp(a)] lowering agents. Panel **B**: Demonstrates the placebo-adjusted percentage reduction of Lp(a) concentration, stratified by dosing frequency and mechanism of action

ASOs are short, chemically modified single-stranded molecules, typically 16–20 nucleotides in length, that hybridize to complementary mRNA through base pairing. Contemporary GalNAc-conjugated ASOs, including pelacarsen, are designed as gapmers. These gapmers have a central DNA gap flanked by ribose-modified wings, typically 2’-methoxyethyl chemistry, that confer nuclease resistance and enhanced target affinity. Once hybridized to target mRNA, the resulting DNA-RNA heteroduplex is recognized by ribonuclease H1 (RNase H1), which cleaves the RNA strand and releases the intact oligonucleotide to engage additional transcripts (Fig. 1A). This catalytic mechanism permits substantial knockdown from a modest drug exposure. Pelacarsen is directed against a sequence within the KIV-2 repeat and exploits the high copy-number structure of this region.

### Small Interfering RNAs

siRNAs are double-stranded RNA molecules, typically 19–23 base pairs in length, that engage the endogenous RNA interference pathway. After cytoplasmic delivery, the duplex RNA molecule is incorporated into RNA-induced silencing complexes (RISC). The catalytic protein Argonaute 2 discards the passenger strand and retains the guide strand, which directs RISC to complementary mRNA for cleavage. The RISC complex is not consumed in this reaction, and a single RISC-loaded guide strand can cleave multiple target transcripts across successive cycles. This is the physical basis for the extended dosing intervals characteristic of the siRNA agents relative to pelacarsen.

Both platforms have demonstrated favorable clinical safety profiles, with injection-site reactions as the most common adverse event and no excess hepatotoxicity or immune activation attributable to the RNA mechanism itself.

### Pelacarsen: The Antisense Oligonucleotide in Phase 3

#### Phase 2 Clinical Data

In a phase 2b, randomized, placebo-controlled dose-ranging trial (NCT03070782) enrolling patients with established ASCVD and Lp(a) above 60 mg/dL, pelacarsen produced dose-dependent and sustained Lp(a) reductions [27]. The 20 mg weekly regimen achieved an 80% mean reduction versus 6% with placebo, with absolute reductions in apoB (-10.9 mg/dL) and LDL-cholesterol (-16.4 mg/dL)(Fig. 1B). Lp(a) levels returned to baseline 16 weeks after the final dose. At the dose subsequently selected for phase 3 testing, 98% of participants achieved Lp(a) below 50 mg/dL. The predominant adverse events were myalgia, arthralgia, and malaise, and a 5% discontinuation rate was attributable to adverse events.

#### Lp(a) HORIZON

Lp(a) HORIZON (NCT04023552) is a global, randomized, placebo-controlled cardiovascular outcomes trial that enrolled 8,323 patients with established cardiovascular disease, defined as prior MI, ischemic stroke, or symptomatic peripheral artery disease, and screening Lp(a) of at least 70 mg/dL, with a prespecified subpopulation at 90 mg/dL or higher [28]. Participants received pelacarsen 80 mg or placebo subcutaneously once monthly on a background of guideline-directed lipid-lowering and antihypertensive therapy. The primary endpoint was a four-point composite of cardiovascular death, non-fatal MI, non-fatal stroke, and urgent coronary revascularization requiring hospitalization, assessed when approximately 993 confirmed primary events had accrued, with a minimum follow-up of 2.5 years per participant (ClinicalTrials.gov identifier NCT04023552, accessed September 4, 2026). The topline results announced in a press release reported that pelacarsen achieved substantial, dose-consistent lowering of Lp(a), but that the trial did not meet its primary composite endpoint in the overall study population compared with placebo [29]. This is the first cardiovascular outcomes trial of any dedicated Lp(a)-lowering agent to report and is therefore a landmark, if sobering, result for the field. Beyond confirmation that the primary endpoint was not met and that Lp(a) was substantially lowered, no further quantitative data, including the magnitude of Lp(a) reduction achieved, the hazard ratio and confidence interval for the primary endpoint, results in the prespecified 90 mg/dL or higher subpopulation, individual endpoint components, or the detailed safety and adverse-event profile, had been disclosed as of this writing. The implications of this result, and the considerable uncertainty that persists pending the complete dataset, are discussed further below. (Table 2)

**Table 2:** Summary of Lp(a)-lowering agents in clinical development

Agent	Platform	Phase	*N*	Primary endpoint	Lp(a) reduction	Trial/NCT	Status
Pelacarsen	ASO (GalNAc)	3	8,323	CV death, MI, stroke, urgent revascularization	80% (phase 2b); magnitude in Lp(a) HORIZON not yet disclosed	Lp(a) HORIZON, NCT04023552	Topline reported Sept. 4, 2026: primary endpoint (4-point MACE) not met despite Lp(a) lowering; full data pending peer-reviewed publication (Novartis, 2026)
Olpasiran	siRNA (GalNAc)	3	7,297	CHD death, MI, urgent revascularization	~ 94–97%	OCEAN(a)-Outcomes, NCT05581303	Active, not recruiting. Estimated completion December 2026 (ClinicalTrials.gov, accessed August 28, 2026)
Zerlasiran	siRNA (GalNAc)	2	178	Percent Lp(a) change	79–86%	ALPACAR-360, NCT05537571	Phase 3 not yet announced (ClinicalTrials.gov, accessed August 28, 2026)
Kylo-11	siRNA (dual GalNAc)	1 complete	70	Safety; Lp(a) change	~ 97% (600 mg, durable to 48 wk)	NCT06363851	Phase 1 complete; phase 2 planned (NCT07327840)
Lepodisiran	siRNA (GalNAc)	3	~ 17,300	MACE composite	~ 95%	ACCLAIM-Lp(a), NCT06292013	Active, not recruiting
Muvalaplin	Oral small molecule	3	~ 10,450	MACE composite	~ 86% (intact assay)	MOVE-Lp(a), NCT07157774	Initiated

Percentage reductions reflect differing endpoint definitions (time-averaged versus single-timepoint) across trials and are not directly comparable; see Comparison of RNA-Based Agents, above. Status current as of September 4, 2026

PVCs. *ASO * antisense oligonucleotide, *siRNA * small interfering RNA, *GalNAc * N-acetylgalactosamine, *MACE * major adverse cardiovascular events.

### Small Interfering RNA Agents: Olpasiran, Zerlasiran, and Lepodisiran

#### Olpasiran

Olpasiran is a GalNAc-conjugated siRNA that has advanced furthest among agents in this class. In the phase 2 OCEAN(a)-DOSE trial, olpasiran produced dose-dependent reductions in patients with established ASCVD and Lp(a) above 150 nmol/L [30]. At the phase 3 dose of 75 mg every 12 weeks, placebo-corrected reduction reached approximately 94% to 97% at week 36, with individual assessments occasionally exceeding 100%, most plausibly reflecting measurement variability as residual concentrations approach the assay’s lower limit of quantification rather than a supraphysiological drug effect. Extension analysis through a median of 86 weeks demonstrated substantial off-treatment durability, with 76–84% suppression sustained at week 60 and 28–36% at week 96, approximately 60–72 weeks after the final dose [31]. A companion analysis found sustained reduction in oxidized phospholipid on apoB without significant change in high-sensitivity C-reactive protein or interleukin-6, suggesting that any eventual clinical benefit is unlikely to be mediated through a systemic anti-inflammatory mechanism [32]. The phase 3 OCEAN(a)-Outcomes trial (NCT05581303) enrolled 7,297 patients with ASCVD and Lp(a) of at least 200 nmol/L randomized to olpasiran 75 mg every 12 weeks or placebo, with the primary endpoint defined as time to coronary heart disease death, MI, or urgent coronary revascularization. Primary completion is estimated for December 2026 (ClinicalTrials.gov identifier NCT05581303, accessed August 28, 2026).

#### Zerlasiran

Zerlasiran is a GalNAc-conjugated siRNA notable for its potent Lp(a)-lowering effect. In a first-in-human single-ascending-dose study, the 300 mg and 600 mg doses produced median maximal reductions of 96% and 98%, respectively, at approximately 150 days [33]. The phase 2 ALPACAR-360 trial evaluated three regimens among patients with stable ASCVD and Lp(a) of at least 125 nmol/L [34]. At week 36, the time-averaged placebo-adjusted reduction reached 86% with 450 mg every 24 weeks. At week 60, 28–36 weeks after the final dose, the 300 mg every-16-week regimen sustained the largest reduction at 79%, consistent with progressive accumulation of RISC-loaded guide strand across successive doses. A dose-dependent, transient rise in C-reactive protein occurred in the first week after injection and resolved without sequelae. The mechanism for this has not been adequately established. As of the most recent published literature, zerlasiran remains the only leading siRNA agent without an announced phase 3 cardiovascular outcomes trial (ClinicalTrials.gov, accessed August 28, 2026).

#### Kylo-11

Kylo-11 is a structurally non-canonical siRNA distinguished by dual GalNAc conjugation, with two moieties at the 3′ terminus of the sense strand and two at the 5′ terminus of the antisense strand, rather than the single 3′-sense-strand conjugation used by olpasiran, zerlasiran, and lepodisiran. In a first-in-human, randomized, double-blind, placebo-controlled phase 1 trial conducted in China, 70 participants received a single subcutaneous dose (9 to 600 mg) or placebo. No injection-site reactions, serious adverse events, or drug-related adverse events occurred at any dose. At doses of 225 mg or higher, Lp(a) reductions were durable through 48 weeks, reaching a median of 97% at the 600 mg dose and 96% in a high-baseline-Lp(a) cohort (Lp(a) greater than 200 nmol/L) given 225 mg. The reductions at lower doses (9 to 75 mg) waned over the same interval [35]. If replicated in larger trials, the dual-GalNAc design and the complete absence of injection-site reactions would distinguish Kylo-11 from the other siRNA agents and could support even less frequent dosing than currently achieved with lepodisiran. A phase 2 trial with sequential dosing is planned (NCT07327840, accessed August 28, 2026).

#### Lepodisiran

Lepodisiran offers the longest duration of action among the siRNA agents. In the phase 1 single-ascending-dose study, a 608 mg dose produced a median reduction exceeding 90% sustained beyond 48 weeks after a single injection. The phase 2 ALPACA trial randomized 320 participants across five regimens. The primary endpoint of placebo-adjusted time-averaged change from day 60 to 180 reached 95% with two 400 mg doses administered six months apart [36]. One year after the second dose, participants receiving two 400 mg doses retained a 74% reduction, and those receiving a single dose retained 53%, eighteen months after their only injection. Injection-site reactions occurred in 11.6% of the two-dose cohort versus 1.4% with placebo.

ACCLAIM-Lp(a) (NCT06292013) is the only announced phase 3 program to include a primary prevention stratum, comprising individuals aged 55 years or older with documented arterial disease but no prior cardiovascular event, alongside a secondary prevention cohort, with Lp(a) of at least 175 nmol/L required for either group. The reported projected enrollment has grown over the course of the trial’s development, reaching approximately 17,300 participants in the most recent published tabulation (ClinicalTrials.gov identifier NCT06292013, accessed August 28, 2026). Given that most of the estimated 1.4 billion individuals worldwide with elevated Lp(a) have not experienced a cardiovascular event, this stratum addresses a substantially larger share of the global attributable burden than the secondary-prevention-only designs of the pelacarsen and olpasiran outcomes trials.

### Comparative Efficacy, Safety, and the Broader Therapeutic Landscape

#### Comparison of RNA-Based Agents

Direct head-to-head comparison of the RNA-based agents has not been performed, and reported percentages are not directly comparable across trials without accounting for the reporting convention used. Because GalNAc-conjugated oligonucleotides are cleared from plasma within hours while pharmacodynamic effect persists for months, a nadir value obtained shortly after dosing overstates the average exposure experienced across a full dosing interval, particularly for agents dosed every 16 to 24 weeks. The ALPACAR-360 and ALPACA investigators addressed this by adopting time-averaged percentage change as the primary endpoint, an approach not uniformly applied across the earlier literature.

With this caveat, several patterns remain informative. All five GalNAc-conjugated agents achieve reductions substantially exceeding existing therapies, ranging from approximately 80% for pelacarsen to more than 90% for the siRNA agents, including Kylo-11. These differences reflect both the pharmacodynamic distinction between RISC persistence and RNase H1 recycling and the specific chemical design of each molecule. The siRNA agents’ prolonged RISC residence confers longer per-dose duration of effect, permitting quarterly to semiannual dosing, and potentially annual dosing with Kylo-11’s dual-GalNAc design, compared with pelacarsen’s monthly requirement.

#### Safety

Safety profiles have been reassuringly consistent across the RNA-based agents in published phase 1 and 2 data, though the cumulative phase 1 and 2 experience across all five agents remains under one thousand participants with follow-up generally measured in months for most agents. Injection-site reactions are the most frequently reported adverse event across the class and have been mild without systemic manifestation. Hepatic and renal function abnormalities have not exceeded background rates, plausibly reflecting the 10- to 30-fold reduction in systemic exposure conferred by GalNAc conjugation relative to earlier unconjugated oligonucleotide chemistries.

A numerical excess of hyperglycemia and new or worsening diabetes was observed with olpasiran during off-treatment follow-up in OCEAN(a)-DOSE (8.8% versus placebo, compared with a more balanced 7.0% versus 5.6% during the 48-week on-treatment period), and this signal has been flagged for prospective adjudication in the phase 3 program [31]. A similar association was reported in a post hoc analysis of ODYSSEY OUTCOMES, in which alirocumab-associated Lp(a) reduction was associated with a higher risk of incident type 2 diabetes [37], consistent with MR data linking constitutively low Lp(a) to diabetes risk, though the causal direction of that relationship remains contested [38–40]. Whether pharmacological Lp(a) suppression itself perturbs glucose homeostasis is a distinct question that only prespecified glycemic adjudication in the ongoing outcomes trials, together with the two independent PCSK9-pathway signals now observed, can resolve. Off-target effects on plasminogen are a theoretical concern given the structural homology of apo(a) but have not been observed in human studies to date.

## Beyond RNA Silencing: Muvalaplin and Gene Editing

### Muvalaplin

Muvalaplin is a first-in-class oral small molecule that binds the KIV-8 domain of apo(a) and blocks its noncovalent interaction with apoB-100 and prevents the extracellular particle assembly rather than suppressing *LPA* gene expression [41]. In the phase 2 KRAKEN trial, muvalaplin at 240 mg daily achieved placebo-adjusted reductions of 85.7% using an intact-particle assay and 69.9% using a conventional apo(a)-based assay at 12 weeks [42]. The conventional assays detect free apo(a) no longer incorporated into particles and therefore underestimate muvalaplin’s true effect [43]. A phase 3 outcomes trial, MOVE-Lp(a) (NCT07157774), enrolling approximately 10,450 participants under an eligibility framework similar to that of ACCLAIM-Lp(a), has been initiated. An effective oral agent would substantially expand access relative to injectable therapies.

### Gene Editing

All agents discussed above require repeated administration because they silence, rather than alter, the *LPA* transcript. The gene continues to direct synthesis and the effect of these agents wanes as the drug is cleared. CRISPR-Cas9 gene editing offers a mechanistically distinct approach through permanent disruption of the LPA coding sequence within hepatocytes and provides the opportunity for durable apo(a) suppression from a single treatment. Proof of concept was established in transgenic mice expressing human apo(a) using a hepatocyte-tropic adeno-associated viral vector to deliver Cas9 and a guide RNA to the first kringle domain, achieving near-complete elimination of plasma apo(a) protein within one week [44]. In cynomolgus monkeys, a single dose produced dose-dependent *LPA* editing, with Lp(a) reductions of up to 90% sustained through day 84 at the highest tested dose, and 94% sustained through day 224 at a lower dose in a separate durability cohort [45]. Transient elevations in liver enzymes resolved spontaneously within two weeks. The corresponding human program, CTX320, an in vivo lipid-nanoparticle-delivered CRISPR-Cas9 construct targeting *LPA*, is in an ongoing phase 1 dose-escalation trial. The sponsor has reported Lp(a) reductions of up to 73% in this dose-escalation phase, though these data have been disclosed only in company communications and have not yet been peer-reviewed. A next-generation construct, CTX321, incorporating a refined design is already in development. Direct peer-reviewed human safety data for this platform instead come from CTX310, targeting *ANGPTL3* rather than *LPA*. A phase 1 trial in 15 adults with refractory dyslipidemia produced dose-dependent reductions in ANGPTL3 (up to 79.7%), LDL-cholesterol (up to 48.9%), and triglycerides (up to 55.2%) by 60 days, with infusion reactions in three participants, one transient aminotransferase elevation, and one death (179 days after the lowest dose) adjudicated as unrelated to treatment [46]. Although *ANGPTL3* and *LPA* are distinct targets, this remains the most direct human safety experience with the platform underlying CTX320. A 2026 American College of Cardiology scientific statement on gene-editing therapy in cardiovascular disease specifically identifies hepatocyte-expressed, monogenic lipid disorders, including elevated Lp(a), as among the most immediately tractable applications of this technology, while cautioning that the durability, cost, and ethical implications of a one-time, irreversible intervention warrant deliberate evaluation [47]. Off-target editing and immune responses to the vector, lipid nanoparticle, and Cas9 require longitudinal surveillance because the intervention is permanent and adverse effects cannot be managed by allowing drug effect to wane, as with the reversible RNA-silencing agents.

## Unanswered Questions and Future Directions

### Validating the Lp(a) Hypothesis

The fundamental question of whether pharmacological Lp(a) lowering reduces cardiovascular events was, until September 2026, entirely untested empirically, resting on epidemiological and MR evidence that supports causality but is silent on the magnitude, timing, and reversibility of that effect within a clinical-trial timeframe. The neutral topline result of Lp(a) HORIZON is the field’s first empirical data point, but with only a press release available at the time of writing, any interpretation must be provisional pending data presentation and peer-reviewed publication. Several considerations bear on that interpretation.

First, pelacarsen’s ~ 80% placebo-adjusted Lp(a) reduction in phase 2b was somewhat below the 86–98% achieved by GalNAc-siRNA agents. Whether this quantitative gap, rather than any qualitative limitation of ASO chemistry, contributed to the neutral result cannot be determined from one trial of one agent. OCEAN(a)-Outcomes and ACCLAIM-Lp(a), which enroll at higher thresholds and use siRNAs with deeper, more durable suppression, will help clarify this. No head-to-head or mechanistic data support the inference that ASOs are inherently less effective than siRNAs at equivalent knockdown.

Second, several features of the trial design merit scrutiny once full data are available. The primary endpoint’s inclusion of urgent coronary revascularization, softer and more subjectively ascertained than death, MI, or stroke, could dilute a true effect concentrated in harder outcomes; component-level results will be informative. The 2.5-year minimum follow-up is short relative to the multi-year trajectories over which LDL-lowering benefit typically accrues. Both arms also received guideline-directed background therapy, narrowing the margin for any single intervention to show incremental benefit.

Third, patients already had established cardiovascular disease, so decades of lifelong Lp(a) exposure had already contributed to atherosclerotic burden before treatment began. An intervention started late may be mechanistically less able to reverse accumulated risk than the same reduction sustained from early adulthood. This bears directly on the MR estimates motivating the field. Burgess et al. [48] estimated that a 101.5 mg/dL absolute reduction would be needed to match the benefit of a 38.67 mg/dL LDL-C reduction, while Lamina and Kronenberg [49], attributing the discrepancy to skewed Lp(a) distributions, estimating that 65.7 mg/dL would suffice and another analysis suggested that 50 mg/dL might suffice in secondary prevention [50]. All these estimates derive from primary-prevention cohorts and extrapolate lifelong genetic exposure to a few years of pharmacologic treatment in secondary prevention, an extrapolation now of uncertain validity given Lp(a) HORIZON’s neutral result. A single neutral secondary-prevention trial does not, on its own, overturn genetic and epidemiological evidence for Lp(a) causality established over a lifetime of exposure. However, it does raise the possibility that the effect size achievable by a multi-year pharmacological intervention is smaller than genetic extrapolations implied, or that *LPA*-locus pleiotropy beyond Lp(a) itself contributed to those associations. Differing eligibility thresholds across Lp(a) HORIZON (≥ 70 mg/dL), OCEAN(a)-Outcomes (≥ 200 nmol/L), and ACCLAIM-Lp(a) (≥ 175 nmol/L) may help triangulate this concentration dependence once all three trials report.

Fourth, real-world observational data offers a complementary explanation. Aggressive background LDL-cholesterol-lowering therapy may itself lower Lp(a)-attributable risk, leaving little residual risk for a dedicated agent to decrease. In 273,770 individuals with established ASCVD in the US Family Heart Database, the gradient of recurrent-ASCVD risk across Lp(a) categories was steep among patients receiving no LDL-cholesterol-lowering therapy but was markedly attenuated among those on high-impact LDL-cholesterol-lowering therapy without a PCSK9 inhibitor and essentially flattened among those additionally receiving a PCSK9 inhibitor [51]. This is likely confounded by indication, since absolute recurrent-ASCVD rates were paradoxically highest among patients receiving the most intensive LDL-lowering therapy, but it is still a pattern directly relevant to a trial population that was by design already on optimized LDL-cholesterol lowering regimens.

Several concrete elements of the full dataset will be needed before the field can meaningfully revise its assessment of the Lp(a) hypothesis: (1) whether any signal of harm was observed, particularly given the theoretical concern regarding apo(a)’s structural homology to plasminogen; (2) whether individual components of the composite endpoint or prespecified secondary endpoints showed directionally favorable, if non-significant, trends; (3) whether an achieved-Lp(a) or on-treatment analysis demonstrates a dose-response relationship between the degree of Lp(a) lowering and event reduction, analogous to the achieved-LDL-cholesterol analyses that have historically clarified ambiguous lipid-trial results; and (4) whether the prespecified 90 mg/dL or higher subpopulation, enriched for more extreme Lp(a) elevation, showed a different result than the overall 70 mg/dL or higher population. Until these data are presented, Lp(a) HORIZON is an important but incomplete first test of the Lp(a) hypothesis, and clinical practice should await the full results along with the OCEAN(a)-Outcomes and ACCLAIM-Lp(a) readouts.

### Lp(a), Coronary Plaque Burden, and the Imaging-Outcome Gap

Elevated Lp(a) levels have been associated with increased progression of coronary plaque burden and increased presence of low-density noncalcified plaque and peri-coronary adipose tissue inflammation during serial coronary CT angiography [52]. Elevated Lp(a) is likewise associated with markedly higher MI risk among patients with low-attenuation plaque than among those without it, mediating 73% of the Lp(a)-MI association [53]. A recent meta-analysis has further corroborated these findings across 16 imaging studies (*n* = 19,822) [54]. Despite the impact on coronary plaque burden and progression, thus far none of the outcome trials have incorporated a plaque-imaging endpoint. Therefore, whether Lp(a) suppression favorably alters plaque burden or plaque composition remains unknown.

### Assay Standardization

The coexistence of molar (nmol/L) and mass-based (mg/dL) Lp(a) assays has long complicated cross-study comparison since the conversion between units is confounded by isoform-dependent differences in molecular weight. The 2026 ACC/AHA guideline now recommends measuring Lp(a) with assays that are calibrated in molar units, insensitive to apo(a) isoform size, and traceable to official reference standard materials [14], a recommendation reinforced by empirical data from screening for the Lp(a) HORIZON trial. Among 13,666 patients with Lp(a) measured in both units on the same validated assay platform, a regression-based conversion formula reproduced the trial’s 70 mg/dL and 90 mg/dL eligibility thresholds with 94–97% accuracy, versus 85–92% for the older conversion factor of 2.5 [28]. At the population level, conversion between units is therefore reasonably reliable when both figures are derived from the same validated assay. At the individual level, mean absolute error remained nearly 17 nmol/L, and direct molar measurement, rather than a formula-based conversion, is preferable for individual risk stratification.

### A Pharmacogenomic Approach: Aspirin and rs3798220

An alternative therapeutic logic, modifying the downstream consequences of elevated Lp(a) rather than its concentration, has been proposed for aspirin. Small observational studies, including post hoc analyses of the ASPREE trial and the Women’s Health Study, suggested that aspirin use might be associated with a lower incidence of cardiovascular disease specifically among individuals with Lp(a) at or above 50 mg/dL, without apparent benefit at lower levels. This hypothesis was tested directly in a large multi-cohort study which pooled data from the Atherosclerosis Risk in Communities, Cardiovascular Health, and Multi-Ethnic Study of Atherosclerosis cohorts [55]. No evidence of an Lp(a)-dependent aspirin effect was found in subgroup analyses at either high or low Lp(a). The association between aspirin use and cardiovascular disease incidence did not differ by Lp(a) level. This rigorous replication does not support concentration-guided aspirin use as a near-term strategy and underscores the need for adequately powered replication before an early signal is translated into practice.

### Beyond ASCVD: Calcific Aortic Valve Stenosis

The 2026 AHA/ACC Guidelines and 2022 EAS consensus statement formally recognized elevated Lp(a) as likely causal in calcific aortic valve stenosis, a condition for which no medical therapy currently slows progression [1, 14]. Lp(a) HORIZON includes exploratory assessment of aortic valve outcomes, though these results were not addressed in the September 2026 topline announcement. A dedicated trial, Lp(a)FRONTIERS-CAVS (NCT05646381), is separately evaluating whether pelacarsen slows progression of mild-to-moderate calcific aortic valve stenosis in patients with Lp(a) above 175 nmol/L. A positive signal in either analysis would represent a clinically significant finding given the absence of any existing therapy for this indication, irrespective of how the primary ASCVD endpoint in Lp(a) HORIZON is ultimately interpreted.

### Patient Selection, Equity, and Access

Given that Lp(a) HORIZON was neutral, there is no clear evidence outlining a population amongst whom Lp(a)-lowering confers clear benefit. Thus, any statements about future eligibility remains provisional. However, the ongoing outcome trials implicitly define the ideal candidate profile of individuals with an enriched Lp(a) phenotype, defined by each trial’s threshold (≥ 70 mg/dL, with a ≥ 90 mg/dL subpopulation, in Lp(a) HORIZON; ≥200 nmol/L in OCEAN(a)-Outcomes; ≥175 nmol/L in ACCLAIM-Lp(a)), combined with a significant residual risk not attenuated by intensive LDL-cholesterol-lowering. Furthermore, the ACCLAIM-Lp(a) additionally enrolling a primary-prevention stratum designates another potential population that may potentially benefit from Lp(a) lowering. Hence, clinical practice and patient selection will likely be shaped by the results of the remaining outcome trials, which may in turn inform further phenotypic enrichment strategies to identify the patients most likely to benefit from Lp(a) lowering.

Elevated Lp(a) exhibits meaningful racial and ethnic heterogeneity. Individuals of African ancestry and women have consistently higher mean Lp(a) concentrations than White men. However, the genetic architecture underlying these differences remains incompletely characterized as the majority of published *LPA* genetic data derive from individuals of White European ancestry. Enrollment in the phase 2 programs has been predominantly White and European, and the ALPACA investigators identified low Black enrollment as a limitation of their own trial. Since *LPA* variant frequency and isoform association differ substantially across ancestries, generalizing a treatment effect from one population to another is an empirical assumption. The subcutaneous injectable formulation of most current agents and their anticipated cost raise legitimate access and adherence concerns. The development of an oral agent (muvalaplin) and the infrequent dosing profile of lepodisiran offer partial solutions but their broader implementation will require proactive health-systems engagement as the highest risk populations that will derive the most benefit are also the most under-resourced.

## Conclusions

The causal role of Lp(a) in ASCVD is now supported by genetic evidence of high confidence. The current generation of RNA-based, small-molecule, and gene-editing agents have demonstrated that pharmacological Lp(a) suppression of 80% or greater is achievable, reproducible, and generally well tolerated across early-phase development. Whether this reduction translates into a reduction in cardiovascular events remains the central unresolved question in the field: the first outcomes trial to report, Lp(a) HORIZON, was neutral for its primary endpoint despite substantial Lp(a) lowering, a result that underscores this question is still open rather than settled, and that will require the full Lp(a) HORIZON dataset and the two remaining outcomes trials to resolve. Pending those full results, no Lp(a)-lowering pharmacotherapy can be recommended on the basis of a demonstrated reduction in cardiovascular events. Clinicians should continue to measure Lp(a) once in adults at elevated or uncertain cardiovascular risk, optimize all other modifiable risk factors more aggressively in patients with elevated Lp(a), and consider referral to ongoing trials for eligible individuals.

## Key References

- Blumenthal RS, Morris PB, Gaudino M, et al: 2026 ACC/AHA/AACVPR/ABC/ACPM/ADA/AGS/APhA/ASPC/NLA/PCNA Guideline on the Management of Dyslipidemia: A Report of the American College of Cardiology/American Heart Association Joint Committee on Clinical Practice Guidelines. J Am Coll Cardiol 2026, 87(19):2624-2757.
  - ○ The current, authoritative U.S. guideline issuing Class 1 recommendations for universal one-time Lp(a) screening, cascade testing, and PCSK9 monoclonal antibody use in eligible patients. It establishes the clinical-practice backbone for the epidemiology, current-therapies, and assay-standardization sections of this review.
- Tsimikas S, Karwatowska-Prokopczuk E, Gouni-Berthold I, et al: Lipoprotein(a) Reduction in Persons with Cardiovascular Disease. N Engl J Med 2020, 382(3):244-255.
  - ○ Pivotal phase 2b dose-ranging trial of pelacarsen, establishing the dose-dependent efficacy and safety profile that underpinned the Lp(a) HORIZON outcomes trial, whose neutral topline result is discussed throughout this review.
- Novartis. Novartis announces Lp(a)HORIZON Phase III topline results for pelacarsen in patients with elevated Lp(a) and established cardiovascular disease (CVD) [Press release]. Available at: https://www.novartis.com/news/media-releases/novartis-announces-lpahorizon-phase-iii-topline-results-pelacarsen-patients-elevated-lpa-and-established-cardiovascular-disease-cvd . Accessed on: Sept 4, 2026.
  - ○ The first cardiovascular outcomes trial of a dedicated Lp(a)-lowering agent to report: pelacarsen substantially lowered Lp(a) but did not meet the primary composite endpoint. Issued as this review was being finalized, this topline announcement reframes the manuscript’s central question from hypothetical to partially, though incompletely, answered.
- O'Donoghue ML, Rosenson RS, Gencer B, et al: Small Interfering RNA to Reduce Lipoprotein(a) in Cardiovascular Disease. N Engl J Med 2022, 387(20):1855-1864.
  - ○ First phase 2 demonstration that a GalNAc-conjugated siRNA can nearly abolish circulating Lp(a). It establishes the dose-response and safety data supporting the OCEAN(a)-Outcomes trial.
- Nissen SE, Ni W, Shen X, et al: Lepodisiran - A Long-Duration Small Interfering RNA Targeting Lipoprotein(a). N Engl J Med 2025, 392(17):1673-1683.
  - ○ Demonstrates durable Lp(a) suppression exceeding one year after a single or double dose, the pharmacodynamic basis for the once- or twice-yearly dosing intervals discussed in this review and for the design of the ACCLAIM-Lp(a) outcomes trial.
- Sarraju, A., Du, X., Zhou, L., Laffin, L. J., Wolski, K., Gu, Y., Liu, Z., Zhang, Q., Cui, K., Yong, X., & Nissen, S. E. (2026). Safety and lipoprotein(a)-lowering effects of Kylo-11, a non-canonical, long-duration small interfering RNA targeting lipoprotein(a): A first-in-human, randomised, double-blind, placebo-controlled, phase 1 trial. Lancet. Advance online publication. https://doi.org/10.1016/S0140-6736(26)01484-4 .
  - ○ The most recently published Lp(a)-directed siRNA trial, introducing a dual-GalNAc conjugation strategy that achieved durable suppression without injection-site reactions. It directly informs the platform-comparison framework central to this review.
- Nicholls, S. J., Ni, W., Rhodes, G. M., Nissen, S. E., Navar, A. M., Michael, L. F., et al. (2025). Oral muvalaplin for lowering of lipoprotein(a): A randomized clinical trial. JAMA, 333(3), 222-231.
  - ○ Phase 2 data for the first oral Lp(a)-lowering agent, illustrating both a mechanistically distinct approach (assembly inhibition rather than synthesis suppression) and an assay-dependent efficacy discrepancy discussed in the measurement section of this review.
- Laffin LJ, Nicholls SJ, Scott RS, et al: Phase 1 Trial of CRISPR-Cas9 Gene Editing Targeting ANGPTL3. N Engl J Med 2025, 393(21):2119-2130.
  - ○ The most direct available peer-reviewed human safety experience with the lipid-nanoparticle-delivered CRISPR-Cas9 platform that underlies the LPA-directed CTX320 program, providing a real-world safety reference for the gene-editing section of this review.
- Ambardekar AV, Bhatt A, Hoekstra M, Kelly MA, Musunuru K, Natarajan P: Gene Editing Therapy in Cardiovascular Disease: 2026 ACC Scientific Statement: A Report of the American College of Cardiology. J Am Coll Cardiol 2026.Advance online publication.
  - ○ The first ACC scientific statement addressing gene-editing therapy in cardiovascular disease. It identifies hepatocyte-expressed monogenic lipid disorders, including elevated Lp(a), as the most tractable near-term application and frames the ethical and durability considerations discussed in this review's gene-editing section.
- Colantonio LD, Wang Z, Ghazi L, et al: Aspirin Use and the Risk for Cardiovascular Disease by Lipoprotein(a) Levels: A Multi-Cohort Study. Eur J Prev Cardiol 2025.
  - ○ A rigorous multi-cohort replication, coauthored by one of us, that found no evidence of an Lp(a)-dependent aspirin effect, superseding smaller post hoc analyses and directly shaping this review's pharmacogenomic-aspirin discussion.
- Burgess S, Ference BA, Staley JR, et al: Association of LPA Variants With Risk of Coronary Disease and the Implications for Lipoprotein(a)-Lowering Therapies: A Mendelian Randomization Analysis. JAMA Cardiol 2018, 3(7):619-627.
  - ○ This study provides MR estimates of the absolute Lp(a) reduction required for clinical benefit which is central to this review's discussion of why trial eligibility thresholds, rather than percentage reduction, may prove decisive.
- MacDougall DE, Tybjaerg-Hansen A, Knowles JW, et al: Lipoprotein(a) and recurrent atherosclerotic cardiovascular events: the US Family Heart Database. Eur Heart J 2025, 46(44):4762-4775.
  - ○ A real-world analysis of >270,000 patients with established ASCVD showing that the excess recurrent-ASCVD risk associated with elevated Lp(a) is markedly attenuated among patients on high-impact LDL-cholesterol-lowering therapy, particularly PCSK9 inhibitors. It offers one observational, hypothesis-generating explanation for the neutral Lp(a) HORIZON result discussed in this review.

## Data Availability

No datasets were generated or analysed during the current study.
